# A multicenter validation and calibration of automated software package for detecting anterior circulation large vessel occlusion on CT angiography

**DOI:** 10.1186/s12883-025-04107-6

**Published:** 2025-03-10

**Authors:** Kyu Sun Yum, Jong-Won Chung, Sue Young Ha, Kwang-Yeol Park, Dong-Ick Shin, Hong-Kyun Park, Yong-Jin Cho, Keun-Sik Hong, Jae Guk Kim, Soo Joo Lee, Joon-Tae Kim, Woo-Keun Seo, Oh Young Bang, Gyeong-Moon Kim, Myungjae Lee, Dongmin Kim, Leonard Sunwoo, Hee-Joon Bae, Wi-Sun Ryu, Beom Joon Kim

**Affiliations:** 1 Department of Neurology, College of Medicine, Chungbuk National University Hospital, Chungbuk National University, Cheongju, Republic of Korea; 2 Department of Neurology, Samsung Medical Center, Sungkyunkwan University College of Medicine, Seoul, Republic of Korea; 3 Artificial Intelligence Research Center, JLK Inc, Seoul, Republic of Korea; 4 Department of Neurology, Seoul National University Hospital, Seoul National University College of Medicine, Seoul, Republic of Korea; 5 Department of Neurology, Chung-Ang University College of Medicine, Chung-Ang University Hospital, Seoul, Republic of Korea; 6 Department of Neurology, Inje University Ilsan Paik Hospital, Inje University College of Medicine, Goyang, Republic of Korea; 7 Department of Neurology, Daejeon Eulji Medical Center, Eulji University School of Medicine, Daejeon, Republic of Korea; 8 Department of Neurology, Chonnam National University Hospital, Chonnam National University Medical School, Gwangju, Republic of Korea; 9 Department of Radiology, Seoul National University Bundang Hospital, Seoul National University College of Medicine, Seongnam, Republic of Korea; 10 Department of Neurology, Seoul National University College of Medicine, Seongnam, Republic of Korea; 11 Cerebrovascular Disease Center, Seoul National University Bundang Hospital, Seongnam, Republic of Korea

**Keywords:** Artificial intelligence, Computed tomography angiography, Large vessel occlusion, Ischemic stroke

## Abstract

**Purpose:**

To validate JLK-LVO, a software detecting large vessel occlusion (LVO) on computed tomography angiography (CTA), within a multicenter dataset.

**Methods:**

From 2021 to 2023, we enrolled patients with ischemic stroke who underwent CTA within 24-hour of onset at six university hospitals for validation and calibration datasets and at another university hospital for an independent dataset for testing model calibration. The diagnostic performance was evaluated using area under the curve (AUC), sensitivity, and specificity across the entire study population and specifically in patients with isolated middle cerebral artery (MCA)-M2 occlusion. We calibrated LVO probabilities using logistic regression and by grouping LVO probabilities based on observed frequency.

**Results:**

After excluding 168 patients, 796 remained; the mean (SD) age was 68.9 (13.7) years, and 57.7% were men. LVO was present in 193 (24.3%) of patients, and the median interval from last-known-well to CTA was 5.7 h (IQR 2.5–12.1 h). The software achieved an AUC of 0.944 (95% CI 0.926–0.960), with a sensitivity of 89.6% (84.5–93.6%) and a specificity of 90.4% (87.7–92.6%). In isolated MCA-M2 occlusion, the AUROC was 0.880 (95% CI 0.824–0.921). Due to sparse data between 20 and 60% of LVO probabilities, recategorization into unlikely (0–20% LVO scores), less likely (20–60%), possible (60–90%), and suggestive (90–100%) provided a reliable estimation of LVO compared with mathematical calibration. The category of LVO probabilities was associated with follow-up infarct volumes and functional outcome.

**Conclusion:**

In this multicenter study, we proved the clinical efficacy of the software in detecting LVO on CTA.

**Supplementary Information:**

The online version contains supplementary material available at 10.1186/s12883-025-04107-6.

## Introduction

Recent advancements in stroke imaging and the development of procedural devices have extended the therapeutic window for endovascular thrombectomy (EVT) in patients with large vessel occlusion (LVO) [1]. Accumulated evidence has redefined the standard of care for LVO patients presenting within 6 to 24 h of their last known well time [2, 3]. Because of limited access to the advanced imaging techniques globally, such as magnetic resonance (MR) perfusion or computed tomography (CT) perfusion imaging [4], recent trials have shed light on more readily available imaging techniques, such as CT angiography (CTA) [5, 6].

Although CTA is primarily utilized for EVT decision-making, swift and accurate interpretation of CTA remains challenging in most emergency rooms without vascular experts, where two-thirds of EVT candidates are routed [7]. Even within comprehensive stroke centers, enhancing the ability to screen CTA for LVO could improve procedural efficiency, optimize staffing, and reduce the time from patient arrival to treatment initiation. With the advent of deep learning, several software packages for detecting LVO in CTA are commercially available [8, 9, 10]. To effectively implement the artificial intelligence (AI) software in clinical practice, thorough validation using external data not involved in model training is imperative.

In medical contexts, there is often an imbalance between normal and abnormal data, which hampers deep learning model training [11]. Data augmentation [12] and random under-sampling [13] are common techniques for addressing class imbalance, often improving model performance. However, in cases where augmentation may distort data, model calibration may be considered to compensate for the imbalance [14]. Ensuring confidence calibration for deep learning models using large multicenter datasets enhances the reliability of their predictions, which is crucial for their practical deployment in safety-critical tasks like medical diagnosis [15]. However, the calibration of deep learning algorithm for detecting LVO in CTA has been never attempted.

In this prospective multicenter study from 6 comprehensive stroke centers, we aimed to clinically validate the commercially available automated LVO detection software (JLK-LVO, JLK Inc., Seoul, Korea) [16] in CTA and to calibrate the probability of the deep learning algorithm using real-world data. Additionally, we investigated the clinical implications of these calibrated LVO probability scores in relation to infarct volumes on follow-up diffusion-weighted imaging (DWI) and functional outcomes three months post-ischemic stroke. This may help further extend the clinical applicability of AI software packages.

## Materials and methods

### Study populations

This multicenter study is based on a brain imaging substudy of the ongoing nationwide stroke registry, Clinical Research Collaboration for Stroke in Korea (CRCS-K), which has recruited over 160,000 patients with stroke [17]. We consecutively enrolled patients with ischemic stroke or transient ischemic attack who were admitted within 7 days of symptom onset from April 2022 to April 2023 at five comprehensive stroke centers (Supplementary Fig. S1). To ensure the heterogeneity of the data, we additionally enrolled a consecutive series of patients from January 2021 to March 2022 at Samsung Medical Center, which did not participate in the nationwide stroke registry (Supplementary Fig. S1). Exclusion criteria were (1) CTA performed beyond 24 h of symptom onset, (2) poor image quality or insufficient contrast to analyze, (3) hemorrhagic transformation or brain tumor, and (4) CTA acquired after EVT.

### Ethics

All patients, or their legal representatives if the patient was unable to communicate, provided written informed consent. The study was developed in accordance with the Declaration of Helsinki and approved by the institutional review board of Seoul National University Bundang Hospital [B-2307-841-303].

### Independent validation dataset

To test the model calibration result, we enrolled a consecutive series of patients with ischemic stroke undergoing CTA within 24 h of symptom between February 2022 to November 2023 at another comprehensive stroke center participating nationwide stroke registry. We excluded patients according to the aforementioned criteria.

### Clinical data collection

We retrieved baseline demographic and clinical information for all study participants from a web-based prospective stroke cohort (strokedb.or.kr). The stroke characteristics included the time interval between the onset of symptoms and time of CTA, the National Institutes of Health Stroke Scale (NIHSS) score [18] at admission, and treatment information. The functional status at 3 months after stroke was measured using the modified Rankin Scale (mRS) score [19], which was determined through a structured telephone interview by an experienced physician assistant at each hospital as previously reported [20, 21].

### CTA imaging protocols

CT angiography images were acquired according to standard departmental protocols in each hospital. The scanning parameters were 90 ~ 120 kVp, 60 ~ 376 mAs, 38.4 or 40-mm beam collimation, 0.33 ~ 0.6-second rotation time, and 0.625 ~ 2 mm thickness (Supplementary Table S1). Diffusion-weighted images were acquired using 1.5 or 3.0 T MRI systems (majority [> 95%] of systems are Phillips or Siemens). Slice thickness was 3 ~ 5 mm, spacing between slices 3.3 ~ 6.5 mm, pixel spacing 0.469 ~ 1.375 mm, repetition time 2426 ~ 8800 ms, echo time 64 ~ 108 ms.

### CTA imaging analysis by vascular experts

In the present study, anterior circulation LVO was operationally defined as an arterial occlusion encompassing the intracranial segment of the internal carotid artery (ICA), as well as the M1 and M2 segments of the middle cerebral artery (MCA-M1 and MCA-M2, respectively). The term “intracranial ICA” specifically denotes the segment extending from the petrous part to the bifurcation with the MCA and the anterior cerebral artery (ACA) [22]. The MCA-M1 segment encompasses the stretch from the MCA-ACA bifurcation to the initial branching of the MCA, while the MCA-M2 segment includes the part ascending vertically along the Sylvian fissure from the MCA branching point [22]. In cases where the MCA divided early, a functional classification was utilized whereby the segment closest to the origin was labeled as M1, with subsequent downstream branches classified as M2 [23]. To confirm the presence of LVO, CTA source images, maximum intensity projection (MIP) images, and three-dimensional rendering images were thoroughly examined by two experienced vascular neurologists (W-S.R and S.H), alongside an evaluation of patients’ magnetic resonance imaging (MRI) scans and symptomatic data. In cases of diagnostic discrepancy, a final determination was made by an experienced neuroradiologist (L.S). Along with the presence of LVO, location (ICA, MCA-M1, and MCA-M2), and the side of LVO were recorded. For bilateral occlusions, a true positive was defined when the AI software-generated heatmap was present on both sides, with the smaller side being at least 50% of the larger side. We define acute LVO as LVO relevant to the index stroke, whereas chronic LVO is defined as LVO that is not relevant to the index stroke. Relevant MCA stenosis is defined as moderate to severe stenosis on CTA that are related to infarcts observed on DWIs.

### Deep learning-based software

Source images of CTA with slice thickness between 0.5 ~ 2 mm were fed into the commercially available deep learning-based software (JLK-LVO, JLK Inc., Seoul, Korea) [16, 24]. In brief, an automated algorithm selects slices from source images to construct MIP images. The vessel segmentation involves a 2D U-Net based on the Inception Module [25], trained to segment vessels in axial MIP images. A vessel occlusion detection algorithm follows, involving the combination of vessel masks into a compressed image for training an EfficientNetV2 model [26]. Finally, the model produced LVO score, probability of LVO by the algorithm, and the side of LVO based on comparison of heatmap size between hemispheres.

### Follow-up imaging analysis

Infarct location was categorized as anterior circulation, posterior circulation, and multiple based on the review of follow-up DWI by an experienced vascular neurologist (J-W. Chung). Follow-up DWI within 7 days after CTA were included to analyze the association between LVO score and follow-up infarct volumes. High signal intensity area on b1000 DWI scan were automatically segmented using a validated 3D U-net software package (JLK-DWI, JLK Inc., Seoul, Korea) [27, 28]. The segmented infarct area was meticulously supervised by an experienced vascular neurologist (J-W. Chung), with manual edits applied when necessary to ensure accuracy.

### Probability calibration

We calibrated the LVO probability score using data from six hospitals in two ways. First, we ran a univariate binary logistic regression model with the ground truth label of LVO. In the mode, ground truth label was entered as a dependent variable and LVO probability for the algorithm as an independent variable. After running the model, calibrated LVO probabilities were obtained. Using the ‘pmcalplot’ command in STATA [29], we displayed a calibration plot comparing observed to expected probabilities, using either non-calibrated or calibrated probability scores. Second, we divided patients into ten groups at 10% intervals of non-calibrated LVO probability and assessed the observed frequency of LVO in each group. Subsequently, we arbitrarily categorized patients into four groups based on the observed frequency of LVO. Using the independent validation dataset, we tested the probability calibration results as means of adjusted LVO probability and 4 categorized groups.

### Statistical analysis

Baseline characteristics among participating centers were compared using the ANOVA or Kruskal-Wallis test for continuous variables, and the chi-square test for categorical variables, as appropriate. To validate the accuracy of the software in diagnosing LVO, we computed the AUROC, as well as sensitivity, specificity, PPV, and NPV. A 1000-repeat bootstrap analysis was employed to calculate the 95% confidence intervals (CIs) for all parameters. The AUROC was used in combination with the DeLong method [30] to compute the standard error (SE) of the AUROC. The cutoff for the LVO score used in the analysis was set at 0.5. A true positive was defined when both the presence and side of LVO were concordant between JLK-LVO and the experts’ consensus. If the presence of LVO was correctly identified but the side was incorrect, the case was classified as a false negative. We conducted additional analyses to determine the optimal threshold that would yield the maximum Youden index (sensitivity + specificity − 1). Given that the software is primarily intended for screening LVO, we also computed specificity, PPV, and NPV at a sensitivity level of 0.90. In addition, because clinicians have access to relevant clinical information before conducting CT imaging, we built three binary logistic regression models to detect LVO: one using only the NIHSS score, another using only the LVO score, and a third combining both NIHSS and LVO scores. We then compared the performance of these models using AUROC. Furthermore, we performed the AUROC analysis at each participating center. To test the deep learning algorithm’s ability to detect isolated MCA-M2 occlusion, we reran the analysis for patients with isolated MCA-M2 occlusion, including those without LVO as the control group. After stratifying patients into groups—acute LVO, chronic LVO, isolated MCA-M2 occlusion, relevant MCA stenosis, and no steno-occlusion of MCA—we compared LVO scores using ANOVA with Tukey for multiple comparison. The association between the calibrated LVO groups and infarct volumes on DWI was analyzed using dot plots and ANOVA with Tukey post-hoc comparison in the independent validation dataset. Additionally, the relationship between the calibrated LVO groups and the 3-month mRS score was analyzed using the Cochran–Armitage test in the independent validation dataset. All statistical analyses were performed using STATA software (version 16.0, TX, USA) and MedCalc (version 17.2, MedCalc Software, Ostend, Belgium, 2017). A *P* value < 0.05 was considered statistically significant.

## Results

### Study population

During the study period, a total of 1,391 patients with ischemic stroke or transient ischemic attack were admitted, and 964 (69.3%) underwent CTA in the emergency room. According to the exclusion criteria, 168 patients were excluded, leaving 796 for analysis. The mean age ± SD of the study population was 68.9 ± 13.7 years, and 57.7% were male. LVO was found in 193 (24.2%) patients, and the median interval from last known well to CTA was 5.7 h (IQR 2.5 to 12.1 h). Demographic characteristics were comparable among the participating centers, except for a history of previous stroke (Table 1). However, the intervals from the last known well to CTA, the prevalence of revascularization therapy, and infarct volumes on follow-up DWI were significantly different. Additionally, CT vendors and parameters of CTA varied significantly across participating centers (Supplementary Table S1). For the independent validation dataset, mean (SD) age was 71.0 (12.8) and 58.1% were male (Supplementary Table S2).

**Table 1:** Baseline characteristics of patients in participating centers

	Hospital A (*n* = 116)	Hospital B (*n* = 144)	Hospital C (*n* = 96)	Hospital D (*n* = 85)	Hospital E (*n* = 104)	Hospital F (*n* = 251)	*p*
Age	69.2 ± 12.9	68.7 ± 14.3	69.1 ± 14.0	71.2 ± 13.4	67.7 ± 15.4	68.5 ± 12.9	0.29
Sex, male	60 (51.7%)	84 (58.3%)	52 (54.2%)	45 (52.9%)	65 (62.5%)	153 (61.0%)	0.41
Large vessel occlusion	26 (22.4%)	41 (28.5%)	23 (24.0%)	21 (24.7%)	20 (19.2%)	62 (24.7%)	0.69
Isolated MCA M2 occlusion	6 (5.2%)	6 (4.2%)	1 (1.0%)	5 (5.9%)	5 (4.8%)	16 (6.4%)	0.41
Infarct location							0.026
Anterior circulation	70 (60.3%)	96 (68.1%)	48 (50.5%)	58 (68.2%)	63 (60.6%)	169 (67.9%)	
Posterior circulation	26 (22.4%)	22 (15.6%)	33 (34.7%)	21 (24.7%)	20 (19.2%)	49 (19.7%)	
Multiple	5 (4.3%)	6 (4.3%)	7 (7.4%)	1 (1.2%)	5 (4.8%)	12 (4.8%)	
No lesion	15 (12.9%)	17 (12.1%)	7 (7.4%)	5 (5.9%)	16 (15.4%)	19 (7.6%)	
Initial NIHSS score	3 (0–7)	3 (1–9)	3 (1–8)	5 (1–10)	4 (1–9)	3 (1–8)	0.18^a^
Previous stroke	15 (12.9%)	20 (13.9%)	23 (24.0%)	22 (25.9%)	15 (14.4%)	58 (23.1%)	0.02
Hypertension	70 (60.3%)	91 (63.2%)	66 (68.8%)	60 (70.6%)	77 (74.0%)	153 (61.0%)	0.13
Diabetes	28 (24.1%)	47 (32.6%)	32 (33.3%)	23 (27.1%)	36 (34.6%)	86 (34.3%)	0.39
Atrial fibrillation	27 (23.3%)	30 (20.8%)	23 (24.0%)	26 (30.6%)	27 (26.0%)	46 (18.3%)	0.24
High-risk cardioembolic source	29 (25.0%)	32 (22.2%)	23 (24.0%)	26 (30.6%)	28 (26.9%)	59 (23.6%)	0.66
CT vendor							< 0.001
Philips	116 (100.0%)	3 (2.1%)	0	0	0	0	
GE medical systems	0	140 (97.9%)	0	0	0	250 (99.6%)	
SIEMENS	0	0	96 (100.0%)	82 (96.5%)	0	0	
Toshiba (Canon)	0	0	0	3 (3.5%)	104 (100%)	1 (0.4%)	
Onset to CTA, hr	11.7 (3.2–27.2)	4.8 (2.3–13.1)	11.9 (5.2–23.9)	4.3 (2.0–11.0)	8.6 (2.5–21.5)	7.6 (3.7–13.9)	< 0.001 ^a^
Revascularization therapy							< 0.001 ^a^
Intravenous only	5 (4.3%)	14 (9.7%)	11 (11.5%)	12 (14.1%)	14 (13.5%)	11 (4.5%)	
Endovascular therapy only	11 (9.5%)	15 (10.4%)	5 (5.2%)	13 (15.3%)	6 (5.8%)	27 (11.0%)	
Combined	12 (10.3%)	18 (12.5%)	4 (4.2%)	5 (5.9%)	5 (4.8%)	6 (2.5%)	
Interval between CTA and DWI, hr	1.6 (0.9–2.9)	0.5 (0.2–2.1)	0.8 (0.4–2.5)	1.6 (1.1–2.9)	2.1 (1.2–7.8)	1.7 (1.2–2.7)	< 0.001 ^a^
Infarct volume on DWI, mL	2.8 (0.3–22.0)	1.8 (0.3–10.8)	3.4 (1.1–32.5)	0.5 (0.1–3.1)	0.6 (0.1–6.2)	4.5 (0.5–35.6)	< 0.001 ^a^

^a^Kruskal-Wallis test was used. Data are presented as mean ± standard deviation, median (interquartile range), or number (percentage). MCA = middle cerebral artery; NIHSS = National Institute of Health Stroke Scale; CTA = CT angiography; DWI = diffusion-weighted imaging

### Performance of automated LVO detection software

**Fig. 1:**
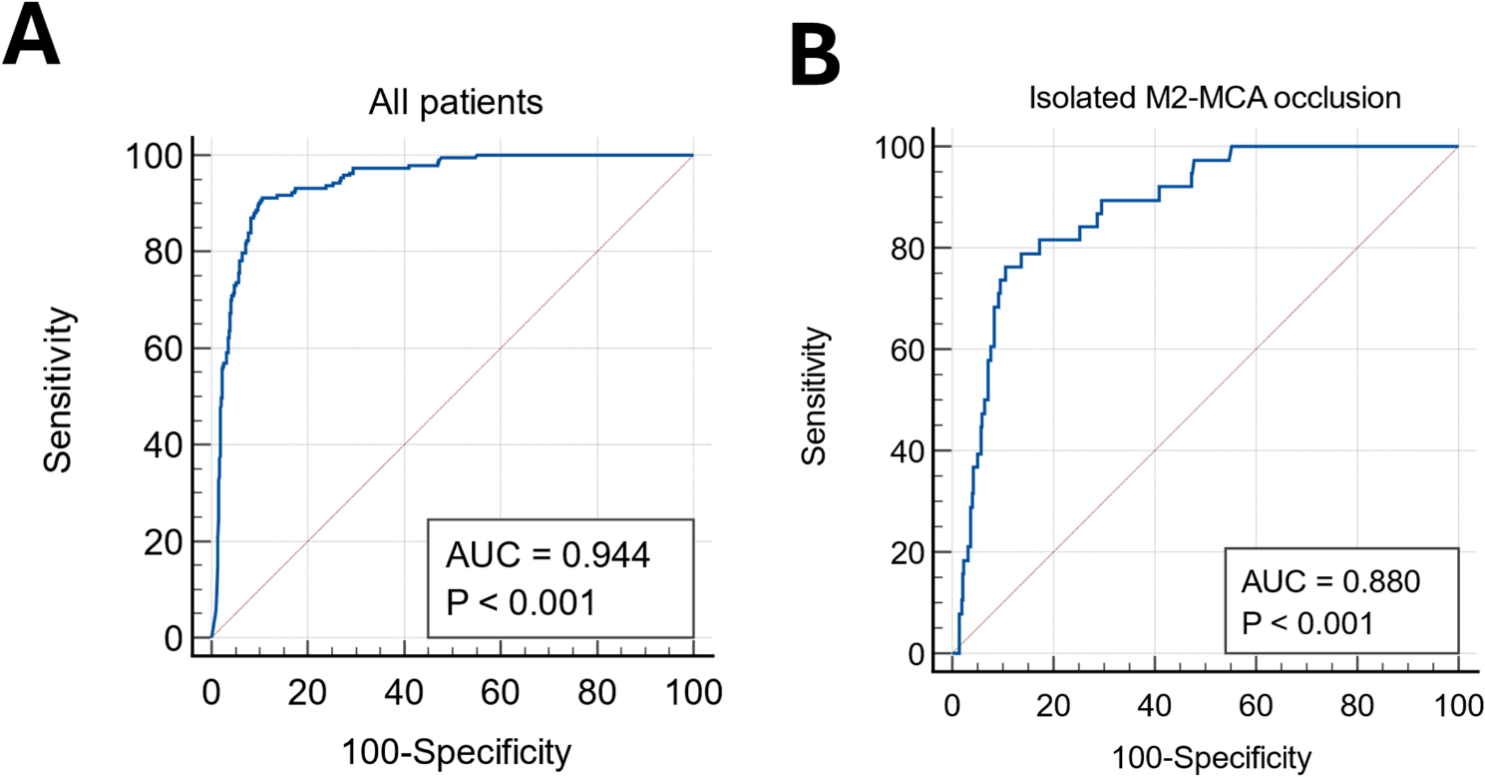
Diagnostic performance of JLK-LVO in the entire population. (**A**) Overall diagnostic performance, (**B**) diagnostic performance in patients with isolated MCA-M2 occlusion

Histogram of LVO score stratified by the presence of LVO showed that the algorithm clearly differentiates LVO from non-LVO (Supplementary Fig. S2). The software achieved an AUROC of 0.944 (95% CI, 0.926–0.960; Fig. 1a) at a cutoff point of 0.50 for the entire population. The sensitivity, specificity, PPV, and NPV were 89.6%, 90.4%, 74.9%, and 96.5%, respectively (Table 2). The highest Youden index was observed at the optimal cutoff point of 0.405, with corresponding values of 91.2% for sensitivity, 89.4% for specificity, respectively. At a fixed sensitivity of 0.90, the specificity was 90.2%. In each participating center, AUROC ranged from 0.913 to 0.970 (Supplementary Fig. S3). When restricted the analysis in patients with isolated MCA-M2 occlusion, AUROC was 0.880 (0.824–0.921, Fig. 1b). At the highest Youden index (0.657; 95% CI, 0.514–0.764), the optimal criterion, sensitivity, and specificity were 0.405, 76.3%, and 89.4%, respectively. The NIHSS-only model achieved an AUROC of 0.819 (95% CI, 0.785–0.853; Supplementary Fig. 4). The LVO-only model yielded an AUROC of 0.939 (0.922–0.957), which was significantly higher than that of the NIHSS-only model. When both NIHSS and LVO scores were incorporated into the model, the AUROC increased to 0.959 (0.947–0.971), which was significantly higher than the other two models.

**Table 2:** Diagnostic performance of software detecting large vessel occlusion

Threshold of 0.50	Confusion matrix	Prediction	
		LVO	No LVO
	Ground truth, LVO	173	20
	Ground truth, no LVO	58	545
	Sensitivity (95% CI)	0.896 (0.845–0.936)	
	Specificity (95% CI)	0.904 (0.877–0.926)	
	PPV (95% CI)	0.749 (0.688–0.804)	
	NPV (95% CI)	0.965 (0.946–0.978)	
Optimal threshold	Youden (J) index (95% CI)	0.806 (0.741–0.843)	
	J_max_ cutoff point	0.405	
	J_max_ Sensitivity	0.912 (0.863–0.948)	
	J_max_ Specificity	0.894 (0.867–0.917)	
Fixed sensitivity of 0.90	Sens_90_ Specificity (95% CI)	0.902 (0.876–0.925)	
	Sens_90_ PPV (95% CI)	0.747 (0.686–0.801)	
	Sens_90_ NPV (95% CI)	0.966 (0.948–0.980)	
	Sens_90_ cutoff point	0.473	

Jmax represents, across all thresholds, the maximum Youden index (sensitivity + specificity − 1). As a secondary reference point, Jmax provides an optimality criterion with equal weighting for sensitivity and specificity. LVO = large vessel occlusion; CI = confidence interval; PPV = positive predictive value; NPV = negative predictive value

### LVO scores according to vessel status

**Fig. 2:**
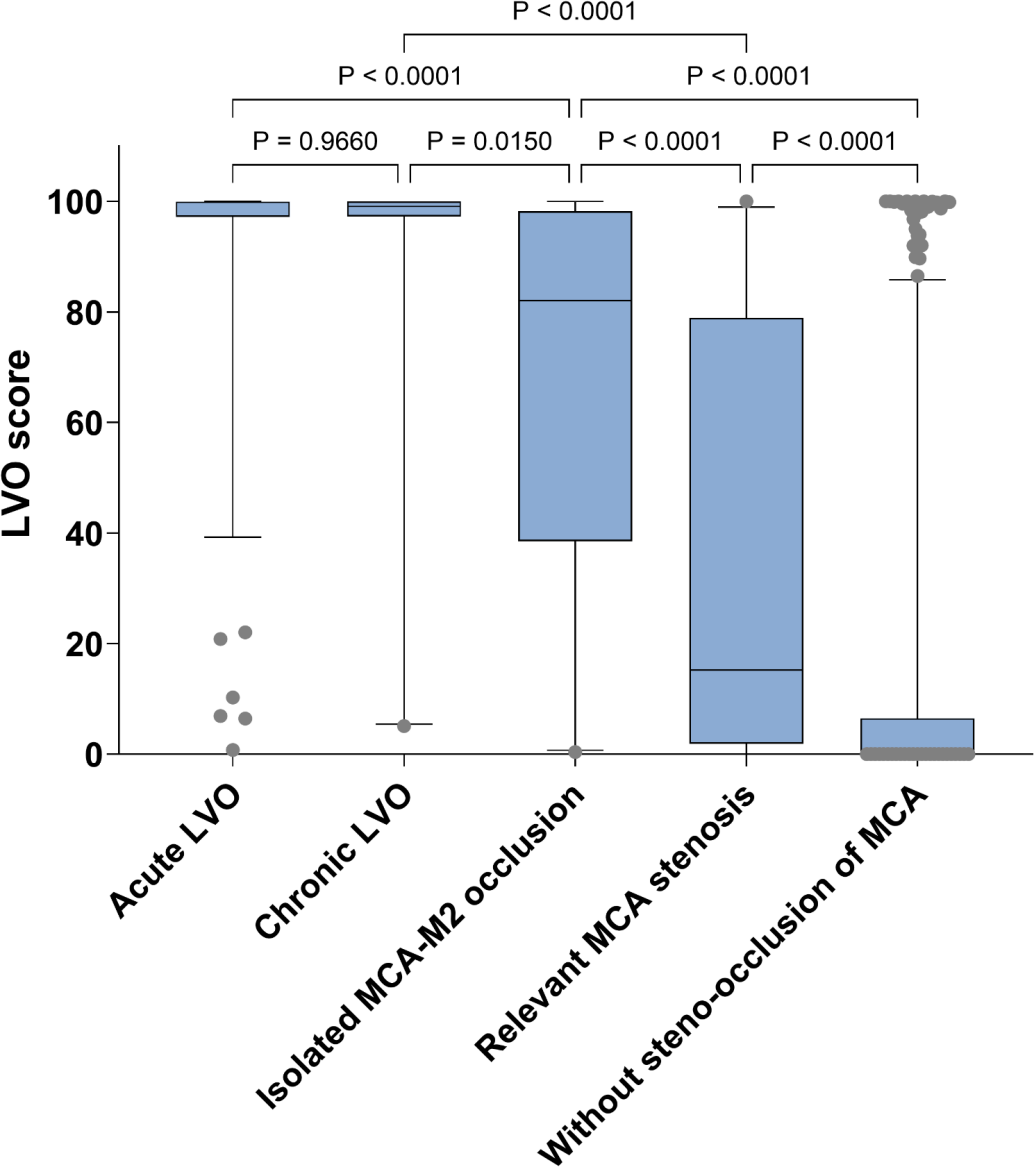
Box plots for LVO score s after stratifying patients according to vessel status. LVO = large vessel occlusion; MCA = middle cerebral artery. Boxes and midline indicate interquartile ranges and the median of LVO scores. Whiskers indicate 5 ~ 95 percentile of data

When stratified patients into five distinct groups (acute LVO, chronic LVO, isolated MCA-M2 occlusion, relevant MCA stenosis, and no steno-occlusion of MCA), the medians (IOR) of LVO scores were 99.8 (97.2–99.97), 99.1 (97.3–99.99), 82.1 (40.9–98.2), 15.3 (2.4–77.4), and 0.5 (0.1–6.5), respectively (Fig. 2). Compared with the no steno-occlusion of MCA group, the median LVO scores of relevant MCA stenosis group was significantly higher (*p* < 0.001).

### Calibration of LVO score

**Fig. 3:**
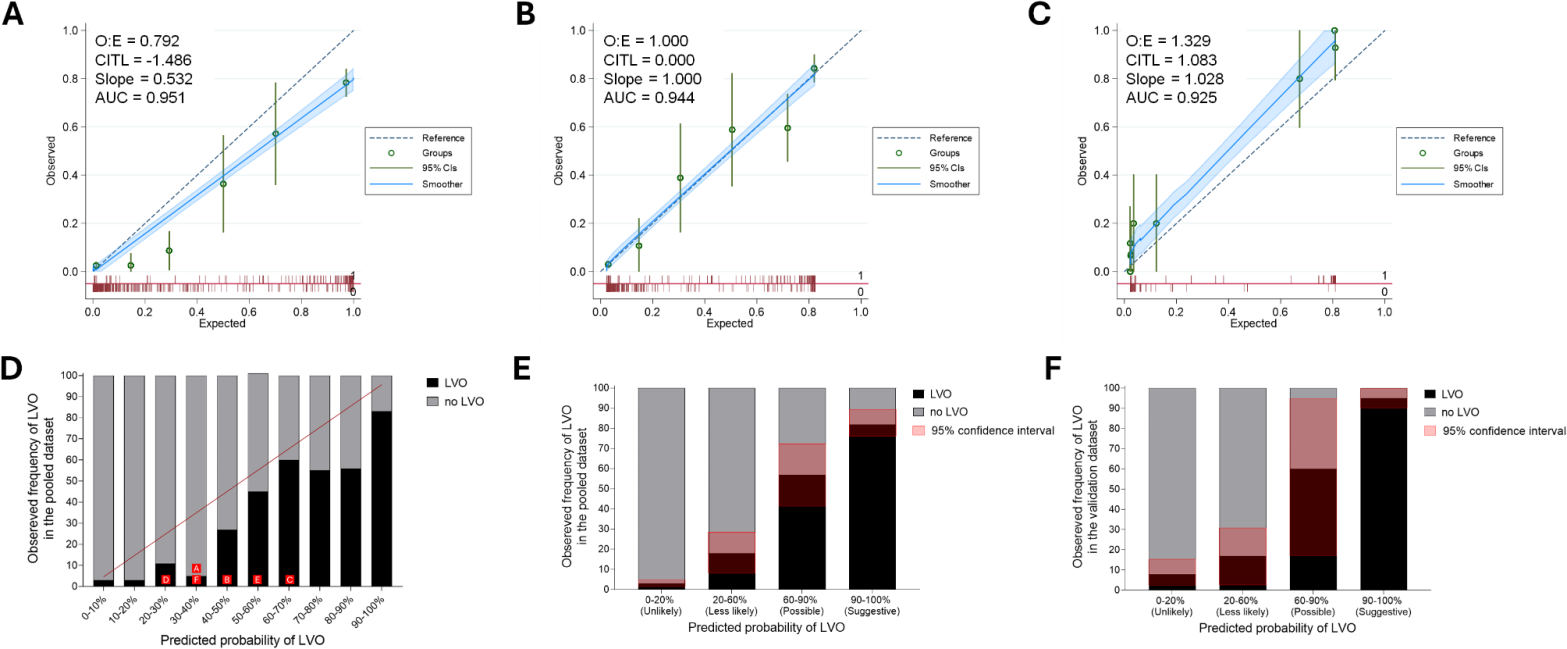
Predicted frequency and observed frequency of LVO before and after recategorization of LVO score. (**A**-**C**) Calibration plots showing observed probability against expected probability using either unadjusted LVO probability (**A**) or adjusted LVO probability (**B**) in the validation and calibration dataset and the independent validation dataset (**C**). The green dotted line indicates the reference line of perfect agreement. Red spikes indicate each case with LVO (up spike) and without LVO (down spike) at each LVO probability. O: E = ratio of observed and expected LVO frequency; CITL = Calibration-in-the-large, also known as mean calibration. (**D**) The red line indicates perfect calibration. The red boxes indicate the criterion with the highest Youden index in each hospital. Observed frequencies and their 95% confidence intervals (red shaded areas) after recategorization of patients in the validation and calibration dataset (**E**) and in the independent validation dataset (**F**). LVO = large vessel occlusion

Non-calibrated LVO probabilities significantly overestimated LVO, as the observed/expected LVO ratio was 0.792 (Fig. 3a). Calibrated LVO probabilities achieved an observed/expected LVO ratio of 1.00 (Fig. 3b). However, due to sparse data between LVO probabilities of 0.2 to 0.6, the point estimations at adjusted probabilities of 0.4, 0.6, and 0.8 exhibited discrepancies between the expected and observed frequencies of LVO even after the calibration. Moreover, in the independent validation dataset, the calibrated probability of LVO underestimated observed frequency of LVO with the observed/expected LVO ratio was 1.329 (Fig. 3c), indicating underestimation of LVO.

As the LVO score percentile increased, the observed frequency of LVO increased in a stepwise manner (p for trend < 0.001; Fig. 3d). However, due to the pronounced bimodal distribution of LVO scores (Supplementary Fig. S2), the observed frequencies of LVO between 20% and 90% showed a low concordance compared to the predicted probability. Based on these results, we arbitrarily recategorized subjects into four groups: unlikely (LVO scores of 0–20), less likely (20–60), possible (60–90), and suggestive (90–100). After the recategorization, each group represented observed frequencies (2.4, 16.9, 56.0, and 79.8%, respectively) well without overlapping confidence intervals (Fig. 3e). In addition, observed frequencies of EVT were 5, 10, 24, and 49% in each group, respectively (Supplementary Fig. S5). In the independent validation dataset, the recategorized group also represented observed frequency of LVO (6.2, 12.5, 60.0, and 90.7%, respectively) although the confidence interval of observed frequency was somewhat wide in the possible LVO group due to small sample size.

### Associations of LVO scores with infarct volumes and functional outcome

**Fig. 4:**
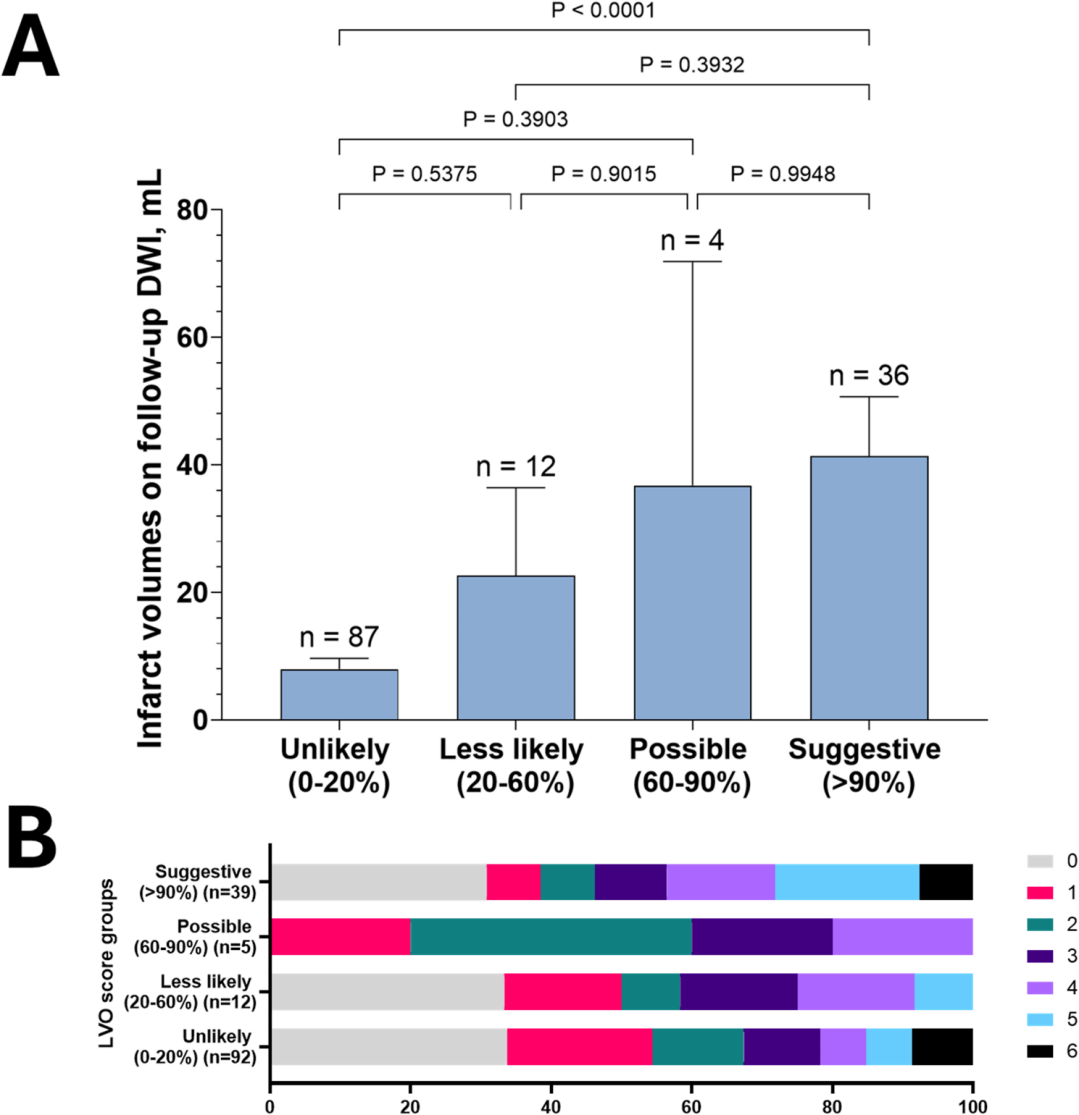
Relation of LVO scores with follow-up infarct volume and functional outcome. (**A**) Bars and error bars represent the mean and its standard error. For post-hoc comparison, the Tukey method was used. (**B**) Distributions of 3-month modified Rankin Scale scores according to LVO score groups

Follow-up DWIs within 7 days of the last known well were available for 139 (93.9%) patients in the independent validation dataset. The median (IQR) interval between CTA and DWI was 2.1 (0.5–57.7) hours. The median (IQR) infarct volumes of the unlikely, less likely, possible, and suggestive groups were 0.9 mL (0.2–5.9 mL), 2.9 mL (1.0–21.0 mL), 2.5 mL (1.1–72.4 mL), and 11.4 mL (0.9–79.8 mL), respectively (p for difference = 0.001; Fig. 4a). Additionally, we observed a significant trend of shifting 3-month mRS scores to higher scores as the recategorized LVO score groups increased (*p* = 0.047; Fig. 4b). A representative case was elaborated in Supplementary Fig. 6.

## Discussion

In this multicenter study comprising 796 consecutive series of patients with ischemic stroke or transient ischemic attack from 6 university hospitals, we observed the robust clinical efficacy of JLK-LVO, an automated software detecting LVO on CTA utilizing a deep learning algorithm. Using a real-world clinical dataset, we calibrated the LVO score derived from deep learning and suggested a new category for better understanding of probability for clinicians. Additionally, we found associations of the new category of LVO score with infarct volumes on follow-up DWI and 3-month modified Rankin Scale scores.

Using a multicenter dataset with various CT vendors and imaging parameters, JLK-LVO exhibited robust and consistent AUROC values ranging from 0.918 to 0.970. The deep learning algorithm in this study was trained on a large dataset of over 2,700 CTA scans from five hospitals [16, 24], enabling it to maintain its performance across different datasets. Additionally, JLK-LVO achieved a sensitivity of 76% in detecting isolated MCA-M2 occlusion, which is comparable to that reported by neuroradiologists in a study involving 520 patients with ischemic stroke; experienced neuroradiologists missed 26% of MCA-M2 occlusions during initial CTA evaluation [31]. Given the recent efforts to expand EVT candidacy to MCA-M2 segment occlusions [32]. the ability to detect MCA-M2 occlusions with high accuracy may facilitate the treatment and benefit of more patients undergoing EVT.

In the present study, we observed a notable bimodal distribution of LVO probabilities score, which, in turn, renders calibration challenging in the range with scarce data. Additionally, different optimal criteria across participating centers suggest that a model-based calibration, commonly used in deep learning algorithms [14], is less practical and prone to miscalibration due to the highly variable disease prevalence and imaging parameters in clinical practice. Hence, we collapsed multiple categories with a similar observed frequency of LVO into one and generated four groups that distinctly represent the observed frequency of LVO. In the independent validation dataset, we showed that the recategorized LVO probability is well correlated with observed frequency of LVO. We believe that this calibrated interpretation, along with uncertainty (the range of observed frequency), provides more reliable results for clinicians.

Of note, LVO scores in patients with relevant MCA stenosis were significantly higher (median 15.3 vs. 0.5) compared to those without MCA stenosis or occlusion. This result indicates that the deep learning algorithm utilizes the symmetry of vascular density between hemispheres as an important feature to detect LVO. Consistent with this finding, we observed an association of LVO score groups with infarct volume on follow-up DWI and 3-month modified Rankin Scale score.

The large size of a consecutive series of CTA data from various vendors and imaging parameters is a strength of our study. Nevertheless, several limitations should be acknowledged. We collected data taken in the emergency room from university hospitals. Hence, further study is required to extrapolate our results to outpatient settings or community hospitals. Additionally, the different head sizes and nature of LVO across ethnicities limited the generalizability of our results [33, 34].

Recent randomized controlled trials [35] have demonstrated the potential of automated algorithms to reduce diagnostic time and improve patient outcomes in acute stroke settings. In this trial, an automated algorithm was applied to segment the ICA terminus and MCA-M1 segments, followed by a comparison of the lengths of the left and right segmentations to identify cases where the ICA terminus and M1 segments were not visible due to ICA occlusion and the absence of retrograde filling. Our approach, which integrates U-Net for vessel segmentation with a deep learning model incorporating the ICA terminus, M1, M2 segments of the MCA, and their branches for LVO prediction, may yield superior results. This is because our algorithm leverages not only the ICA terminus and M1 segment information but also the density of their branches within the MCA territory, providing a more comprehensive analysis.

In conclusion, this multicenter study confirmed the performance of deep learning algorithm for detecting LVO across various CT vendors and imaging parameters. The robust performance of the algorithm, coupled with high accuracy in detecting MCA-M2 occlusions, may enhance stroke workflow, particularly in resource-limited communities. Furthermore, calibrating the LVO probability provides more reliable and interpretable results for clinicians, especially early-career physicians worldwide.

## Electronic supplementary material

Below is the link to the electronic supplementary material.

Supplementary Material 1

## Data Availability

The datasets and code used in this study are available from the corresponding author on reasonable request.

## Abbreviations

LVO: Large vessel occlusion
CTA: Computed tomography angiography
AUC: Area under the curve
MCA: Middle cerebral artery
EVT: Endovascular thrombectomy
CT: Computed tomography
AI: Artificial intelligence
DWI: Diffusion-weighted imaging
NIHSS: National Institute of Health Stroke Scale
mRS: Modified Rankin scale
ICA: Internal carotid artery
ACA: Anterior cerebral artery
MRI: Magnetic resonance imaging
CI: Confidence interval
