# Supplementary material for: A multicenter validation and calibration of automated software package for detecting anterior circulation large vessel occlusion on CT angiography
Source: BMC Neurol. 2025 Mar 10;25:100. doi: 10.1186/s12883-025-04107-6 (PMC11892136; doi:10.1186/s12883-025-04107-6)
Supplement: Supplementary file 1 — Supplementary Material 1 [file 12883_2025_4107_MOESM1_ESM.docx]

**Supplementary Material**

**A multicenter validation and calibration of automated software package for detecting anterior circulation large vessel occlusion on CT angiography**

Yum and Chung et al.

**Figure S1. Study flow chart**


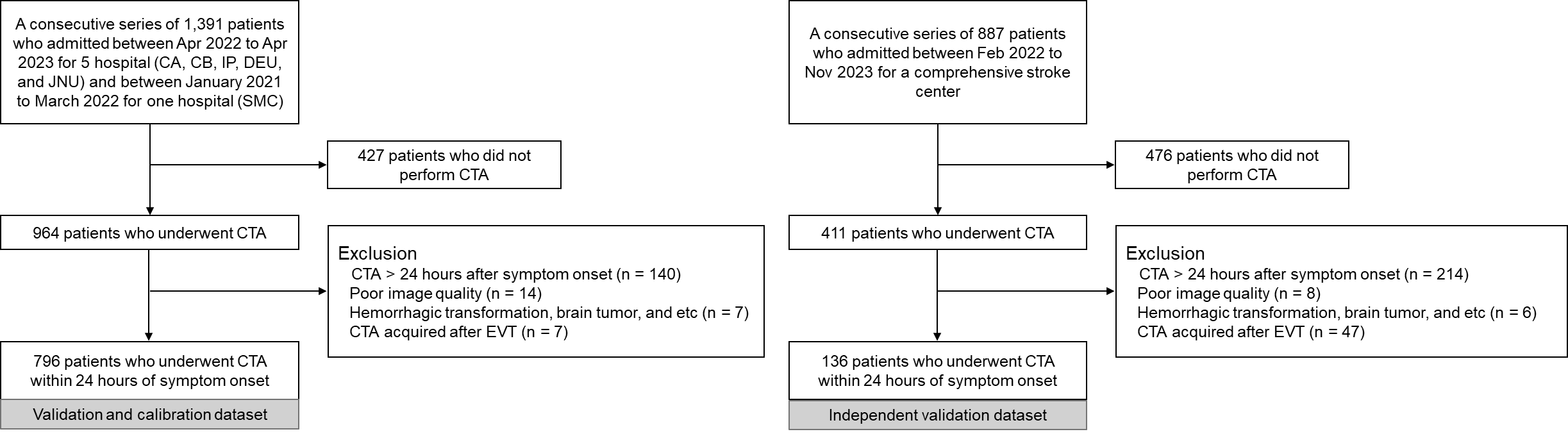


**Figure S2. Histogram of LVO score according to the presence of LVO**

**
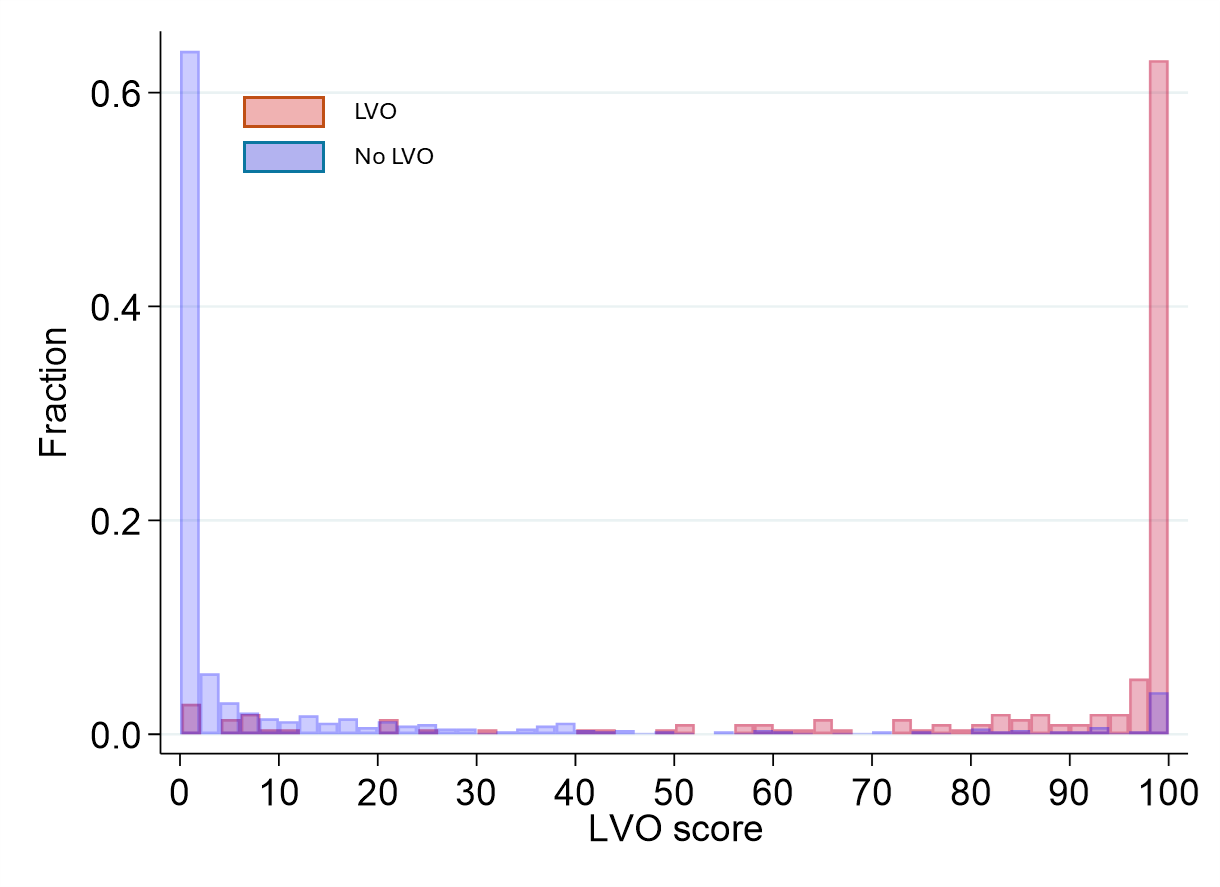
**

**Figure S3. Area under the receiver operating characteristics curve in each participating center**

**
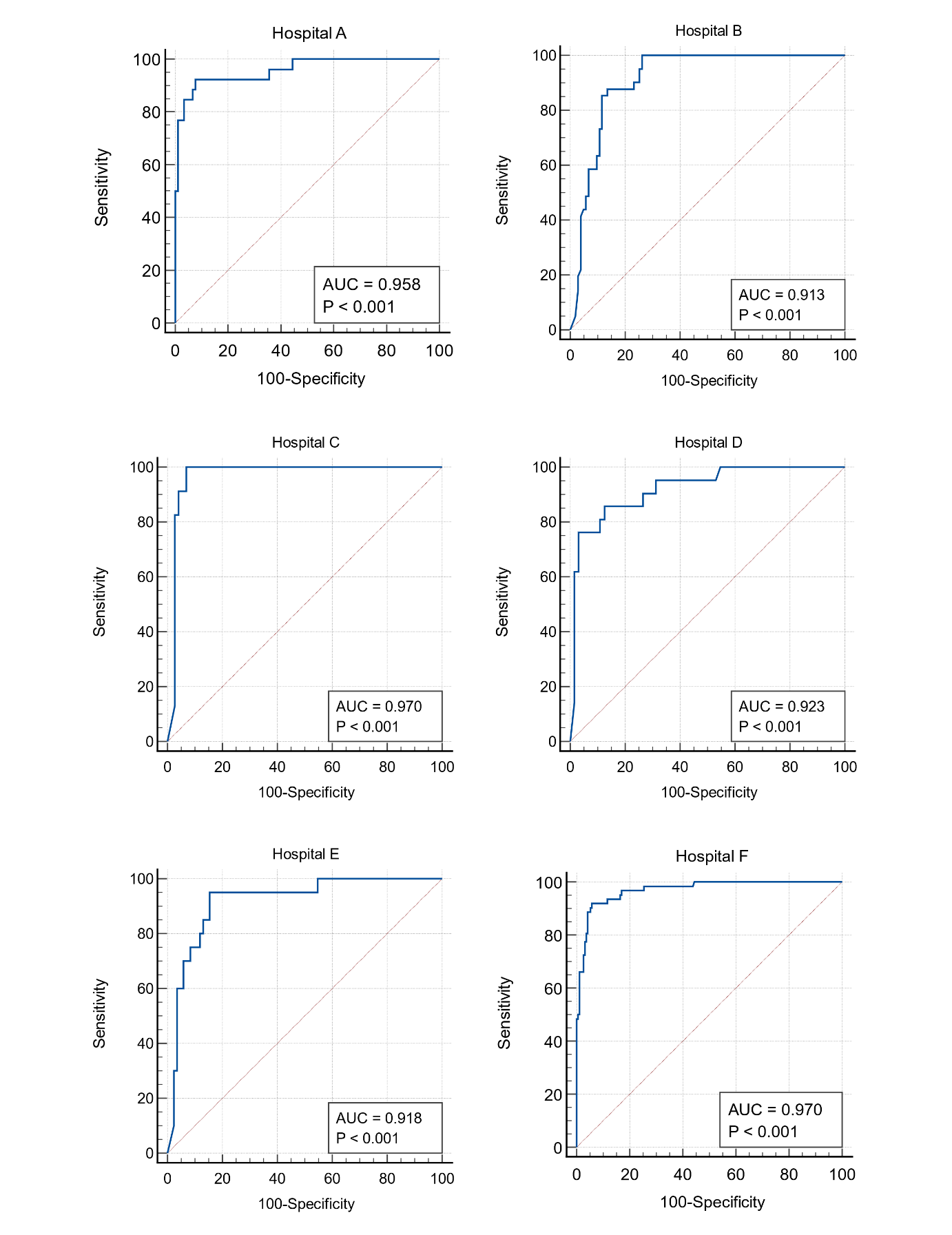
**

**Figure S4. Area under the receiver operating characteristics from 3 different models incorporating NIHSS score only, LVO score only, and NIHSS score + LV score**

**
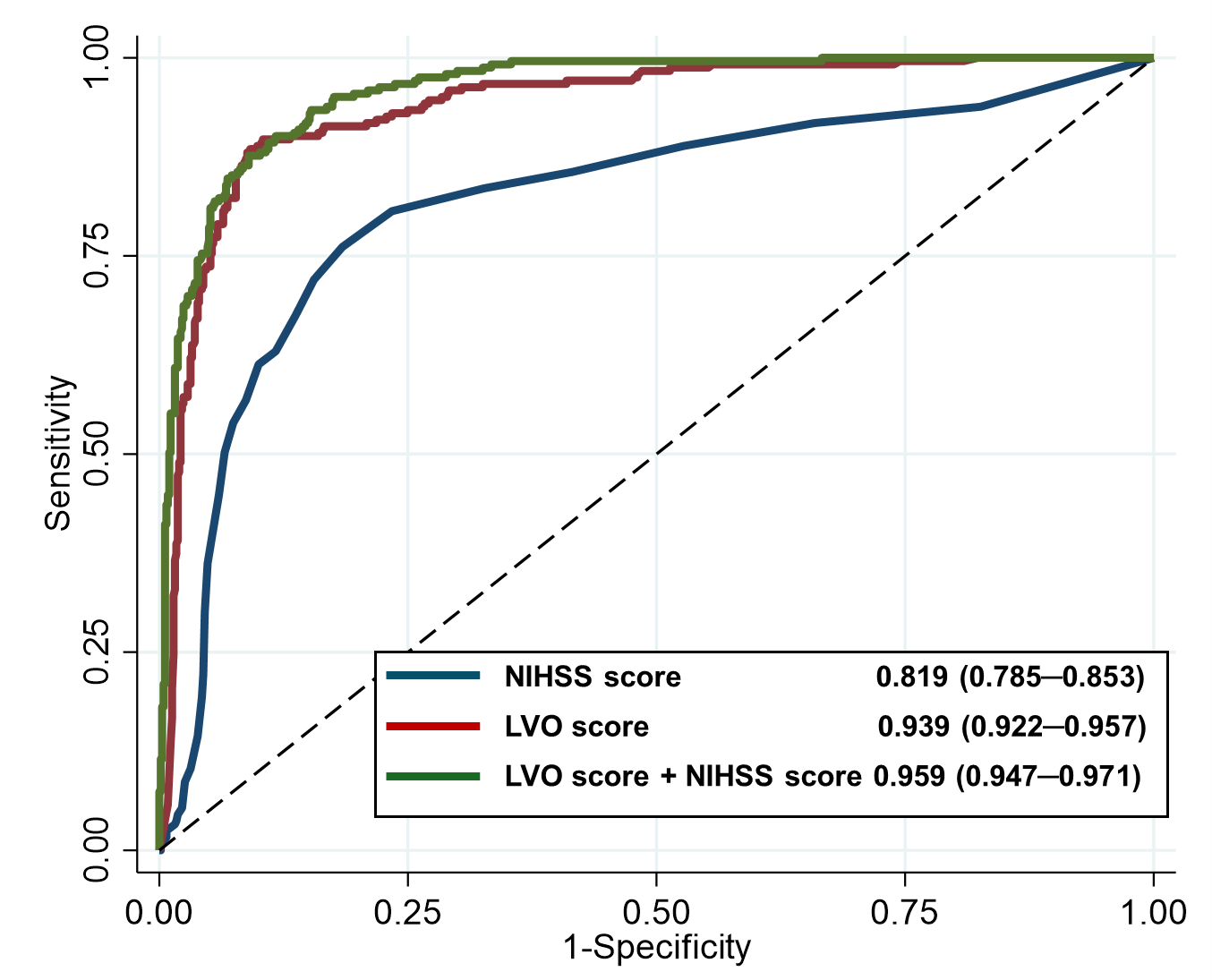
**

Compared with only NIHSS score model, AUROCs of only LVO score and combined NIHSS score an LVO score model were significantly higher (p for difference < 0.001). Compared with LVO score only model, combined NIHSS score an LVO score model were significantly higher (p for difference=0.001). NIHSS=National Institute of Health Stroke Scale; LVO=large vessel occlusion; AUROC=area under the receiver operating characteristics curve.

**Figure S5. Observed frequency of endovascular treatment across LVO score groups**

EVT=endovascular treatment; LVO=large vessel occlusion**.**

**
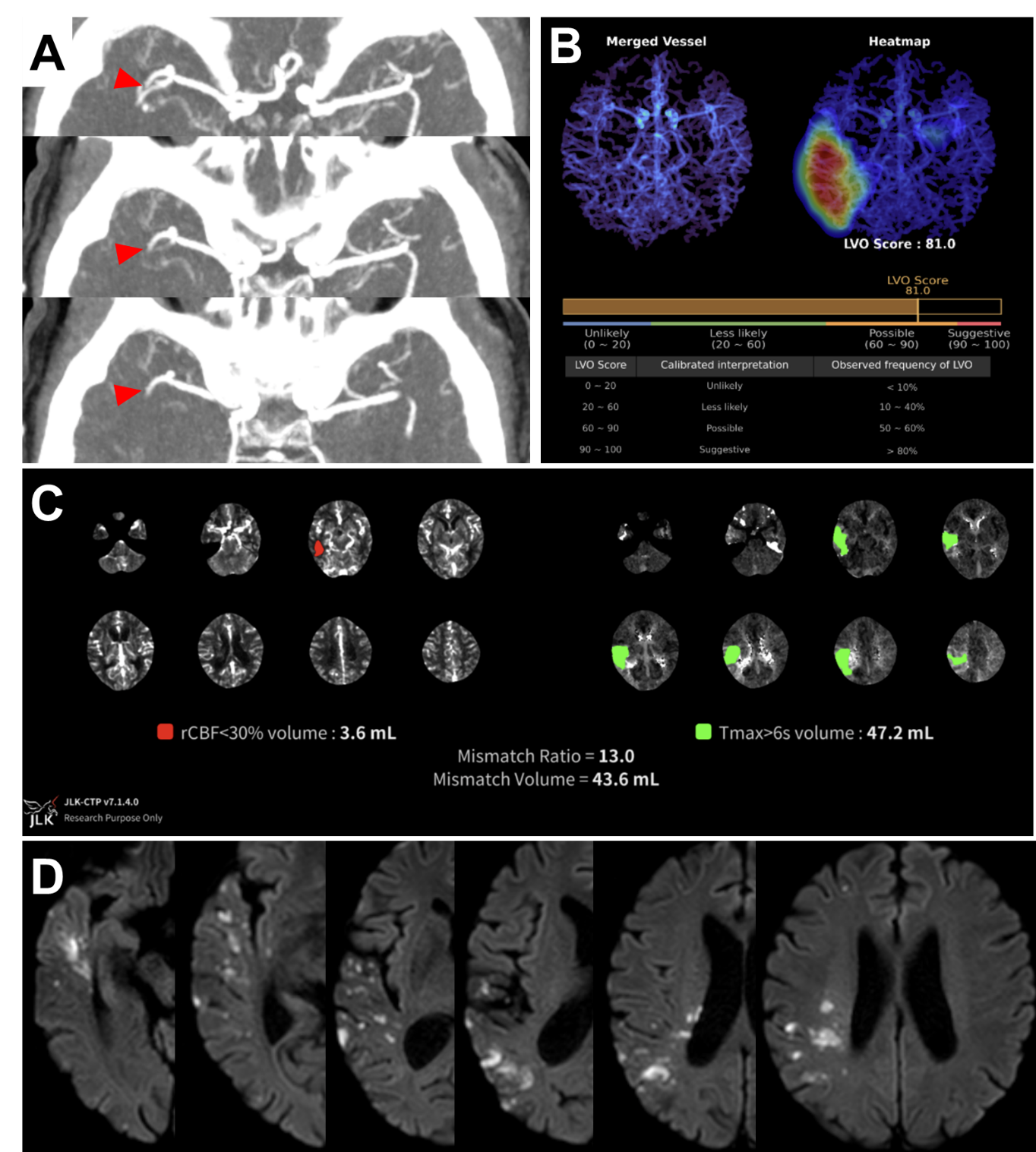
**

**Figure S6. Representative case analyzed by JLK-LVO.** A man in his seventies presented with left-sided weakness and dysarthria, with an initial NIHSS score of 6. (A) Maximum intensity projection image from CT angiography revealed occlusion of the right M2 segment of the middle cerebral artery (red arrowheads). (B) The JLK-LVO score was 81.0, indicating possible large vessel occlusion (LVO), with the heatmap highlighting the distal middle cerebral artery territory. (C) On CT perfusion imaging performed immediately after CT angiography, the ischemic core and hypoperfused areas were measured as 3.6 mL and 47.2 mL, respectively. The patient underwent endovascular thrombectomy. (D) Follow-up diffusion-weighted imaging performed 5 days post-thrombectomy showed diffuse scattered ischemic lesions. The patient achieved a favorable outcome with a 3-month modified Rankin Scale (mRS) score of 1.

**Table S1. CT angiography parameters in participating centers**

|  | Validation and Calibration dataset | | | | | | Independent validation dataset (n = 148) |
| --- | --- | --- | --- | --- | --- | --- | --- |
|  | Hospital A  (n = 116) | Hospital B  (n = 144) | Hospital C  (n = 96) | Hospital D  (n = 85) | Hospital E  (n = 104) | Hospital F  (n = 251) |  |
| Slice thickness, mm | 1.5 | 0.625 | 0.75 | 1.0 | 2 | 1.25 | 1.0 |
| CT manufacture | Phillips | GE medical systems | SIEMENS | SIEMENS | TOSHIBA | GE medical systems | Phillips (n=138)  Siemens (n=10) |
| CT model name | iCT 256 | Revolution CT | SOMATOM Definition AS+, SOMATOM Definition Flash | SOMATOM Definition Edge, SOMATOM Force | Aquilion PRIME | Discovery CT750 HD | Brilliance 64, SOMATOM Force, iCT 256 |
| kVp | 120 | 120 | 120 | 90, 100, or 120 | 120 | 120 | 80, 100, or 120 |
| Rotation time,^a^ s | 0.33 | 0.5 | NA | NA | 0.5 | 0.6 | 0.32 |
| Total Collimation Width, mm | 40 | 40 | 38.4 | 38.4 | 40 | 40 | 40 or 80 |
| Spiral Pitch Factor | 0.515 (n = 62)  0.601 (n = 54) | 0.984375 | 1.2 | 0.45 (n = 26)  0.7 (n = 28)  1.0 (n = 27) | 0.813 | 0.984375 | 0.298~0.789 |
| mAs | 140 (n = 54)  200 (n = 62) | 249.5 ~ 251.5 | 327 ~ 338.5 | 61.75 ~ 330 | 100 (n = 2)  125 (n = 102) | 60 ~ 376.2 | 149~276 |

^a^Rotation time was not available in SIEMENTS CT scanners.

**Table S2. Baseline characteristics of the independent validation dataset (n = 148)**

| Age | 71.0±12.8 |
| --- | --- |
| Sex, male | 86 (58.1%) |
| Large vessel occlusion | 50 (33.8%) |
| Isolated MCA M2 occlusion | 11 (7.4%) |
| Infarct location |  |
| Anterior circulation | 102 (69.4%) |
| Posterior circulation | 28 (19.1%) |
| Multiple | 4 (2.7%) |
| No lesion | 13 (8.8%) |
| Initial NIHSS score | 7 (4 – 16) |
| Previous stroke | 37 (25.0%) |
| Hypertension | 105 (71.0%) |
| Diabetes | 58 (39.2%) |
| Atrial fibrillation | 49 (33.1%) |
| High-risk cardioembolic source | 29 (19.6%) |
| CT vendor |  |
| Philips | 138 (93.2%) |
| GE medical systems | 0 |
| SIEMENS | 10 (6.8%) |
| Toshiba | 0 |
| Canon | 0 |
| Onset to CTA, hr | 3.98 (1.89 – 17.68) |
| Revascularization therapy |  |
| Intravenous only | 42 (28.4%) |
| Endovascular therapy only | 29 (19.6%) |
| Combined | 23 (15.5%) |
